# P-1844. T cell Subtypes and DNA Methylome Dynamics Predict Infection after Kidney Transplantation

**DOI:** 10.1093/ofid/ofae631.2005

**Published:** 2025-01-29

**Authors:** Fei-Man Hsu, Harry Pickering, Monica Cappelletti, Elaine Reed, Matteo Pellegrini, Joanna M Schaenman

**Affiliations:** David Geffen School of Medicine, Los Angeles, California; David Geffen School of Medicine, Los Angeles, California; David Geffen School of Medicine at UCLA, Los Angeles, California; David Geffen School of Medicine at UCLA, Los Angeles, California; David Geffen School of Medicine at UCLA, Los Angeles, California; University of California Los Angeles, David Geffen School of Medicine, Los Angeles, California

## Abstract

**Background:**

Infectious complications continue to cause significant morbidity and mortality after kidney transplantation (KTx), especially in older patients where immune senescence increases impact of immunosuppression. Given the importance of T cell function and of epigenetic regulation in infection vulnerability, we sought to evaluate whether the DNA methylome and T cell phenotypes could predict development of infection after KTx.

**Figure:**
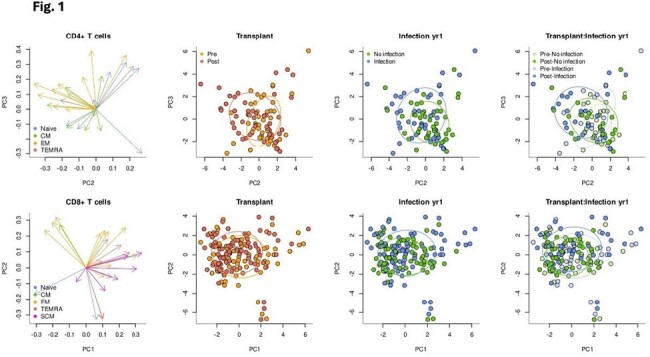

Principal component analysis (PCA) of CD4 and CD8 subsets associated with PBMC collected pre (green) compared with post (blue) transplantation, as well as T cell subsets associated with development of infection (red) versus freedom from infection (orange).

**Methods:**

We evaluated PBMC from 91 KTx patients with samples collected before and then 3 months after transplantation. Deep flow cytometry was performed to delineate naïve, central memory (CM) effector memory (EM), and senescence subtypes including TIM3 and TIGIT in CD4 and CD8 T cells. Targeted bisulfite sequencing (TBS-seq) was applied to PBMC, utilizing probes directed at regions important for T cell maturation, activation, and control of viral infections.

**Figure:**
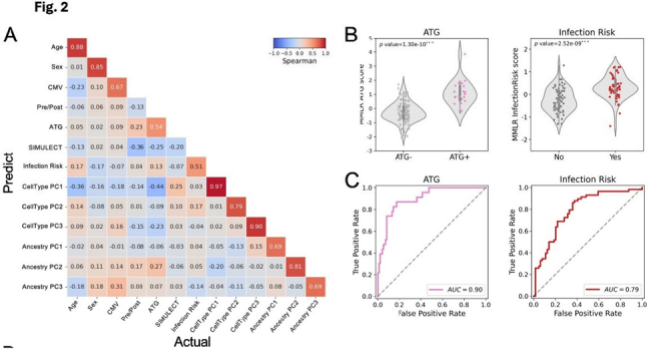

Multivariate multiple linear regression model. A Spearman correlation matrix of actual-prediction traits. B Distribution of predicted values of ATG (left) and infection risk (right). C ROC curves of ATG (left) and infection risk (right).

**Results:**

Transplantation had significant impact on T cell phenotypes, with increase in CD4 memory T cell subtypes CM TIM3+ (p< 0.001) and EM CD127+ (p=0.001) and decrease in CD4 naïve T cells, especially after receipt of antithymocyte globulin (ATG) induction (p=0.008). CD4 T cell subtypes measured pre-transplant were also significantly associated with infection after KTx, including CM (p=0.014) and EM TIGIT (p< 0.001). PCA analysis incorporating multiple maturation subtypes demonstrated ability to separate patients by transplant status and development of infection (**Figure 1**). Differential DNA methylation similarly demonstrated ability to predict infection: Multivariate linear regression identified significant association with infection post KTx with correction for age, sex, CMV serostatus, and ATG induction (**Figure 2A**). An integrated epigenetic risk score was associated with ATG induction and development of infection (**Figure 2B**), with AUC of 0.90 and 0.79, respectively (**Figure 2C).**

**Conclusion:**

Immune markers based on T cell phenotype and differential methylation demonstrate the ability to predict infection after KTx. Pre- and post-transplant immune monitoring has the potential for patient risk stratification and individualization of immunosuppression to improve clinical outcomes by decreasing infection risk after transplantation.

**Disclosures:**

**Joanna M. Schaenman, MD, PhD, FAST**, Eurofins Viracor: Honoraria|F2G: Grant/Research Support|MedCure: Advisor/Consultant|Moderna: Clinical trial support to institution|OneLegacy: Advisor/Consultant

